# Motor symptoms of Parkinson’s disease: critical markers for early AI-assisted diagnosis

**DOI:** 10.3389/fnagi.2025.1602426

**Published:** 2025-07-18

**Authors:** Ni Yang, Jing Liu, Dan Sun, Jiajun Ding, Lingzhi Sun, Xianghua Qi, Wei Yan

**Affiliations:** ^1^Department of First Clinical Medical College, Shandong University of Traditional Chinese Medicine, Jinan, China; ^2^College of Rehabilitation Medicine, Shandong University of Traditional Chinese Medicine, Jinan, China; ^3^Extravascular Department, Qingdao Traditional Chinese Medicine Hospital, Qingdao Hiser Hospital Affiliated of Qingdao University, Qingdao, China; ^4^School of Design, Shanghai Jiao Tong University, Shanghai, China; ^5^Neurology Department, Affiliated Hospital of Shandong University of Traditional Chinese Medicine, Jinan, China

**Keywords:** Parkinson’s disease, motor symptoms, artificial intelligence, diagnosis, markers

## Abstract

Parkinson’s disease is a prevalent neurodegenerative disorder, where early diagnosis is essential for slowing disease progression and optimizing treatment strategies. The latest developments in artificial intelligence (AI) have introduced new opportunities for early detection. Studies have demonstrated that before obvious motor symptoms appear, PD patients exhibit a range of subtle but quantifiable motor abnormalities. This article provides an overview of AI-driven early detection approaches based on various motor symptoms of PD, including eye movement, facial expression, speech, handwriting, finger tapping, and gait. Specifically, we summarized the characteristic manifestations of these motor symptoms, analyzed the features of the data currently collected for AI-assisted diagnosis, collected the publicly available datasets, evaluated the performance of existing diagnostic models, and discussed their limitations. By scrutinizing the existing research methodologies, this review summarizes the application progress of motor symptom-based AI technology in the early detection of PD, explores the key challenges from experimental techniques to clinical translation applications, and proposes future research directions to promote the clinical practice of AI technology in PD diagnosis.

## 1 Introduction

Parkinson’s disease is the second most common neurodegenerative disease worldwide (Elbaz et al., 2016). It is distinguished by motor symptoms such as bradykinesia, resting tremor, rigidity, and postural instability, along with those non-motor symptoms such as decreased sense of smell, sleep disturbances, and cognitive decline (Bloem et al., 2021). According to the Global Burden of Disease study, the prevalence and incidence rates of PD have increased significantly in recent decades. It is estimated that there will be more than 12 million cases worldwide by 2040, representing a huge burden on public health systems (Zhang et al., 2024). Despite the typical clinical manifestations of PD, early diagnosis remains a major challenge. Current diagnostic criteria, such as the UK Brain Bank criteria (Clarke et al., 2016) and the MDS clinical diagnostic criteria (Postuma et al., 2015), rely heavily on subjective clinical assessment. Patients are typically discovered only after the onset of overt motor symptoms. By this time more than 50% of dopaminergic neurons in the substantia nigra may have been lost, missing the best opportunity for early intervention (Lee et al., 2022). Traditional biomarkers, such as cerebrospinal fluid alpha-synuclein assays and dopamine transporter imaging, can improve diagnostic accuracy but are limited by high cost, invasiveness, and accessibility, making them unsuitable for large-scale screening (Bernhardt et al., 2025). Therefore, seeking rapid, objective, non-invasive, and scalable early diagnostic methods is critical.

Recent advances in artificial intelligence (AI), particularly Machine learning (ML) and deep learning (DL), have shown tremendous potential for early detection of PD. Research has shown that subtle and quantifiable preclinical movement disorders occur before the clinical onset of PD, including abnormal eye movements, reduced facial expressions, speech changes, difficulty writing, irregular finger-tapping rhythms, and gait disturbances (Rana et al., 2022a). These features can be captured through computer vision, speech signal processing, and motion sensor analysis, enabling AI-driven automatic detection and classification (Rana et al., 2022b). Compared with traditional diagnostic methods, AI-based recognition has high sensitivity, repeatability, and non-invasive nature, which is helpful for objective screening and remote diagnosis (Gupta et al., 2023). However, there are still some challenges, including data heterogeneity, model interpretability, cross-population generalizability, and clinical feasibility (Voigtlaender et al., 2024).

This review covers early detection of PD using AI in the field of motor symptom-related areas: eye movements, facial expressions, speech, handwriting or finger tapping, and gait analysis. We discussed the specific manifestations of these symptoms, the workflow of AI-assisted diagnosis, data collection methods, feature extraction techniques, available public datasets, and the current performance and limitations of diagnostic models. By evaluating existing research methods, we explored the opportunities and challenges of integrating AI into early PD diagnosis and provided insights for future research to advance the use of AI and precision-driven PD diagnostic systems in clinical practice.

## 2 Motor symptoms and manifestations of PD

**FIGURE 1:**
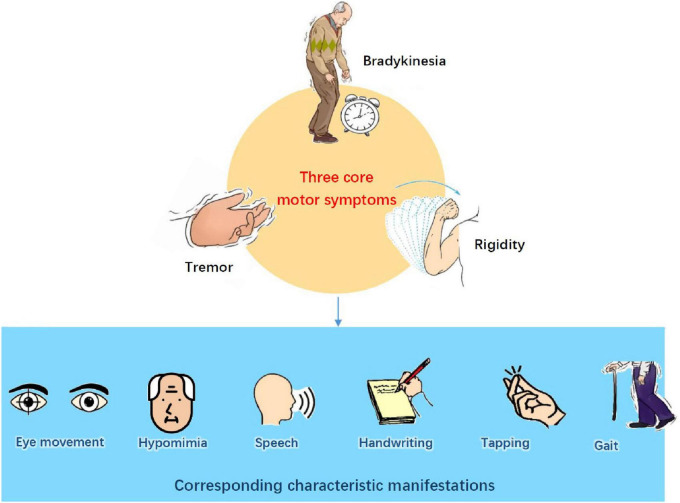
Motor symptoms and corresponding characteristic manifestations of Parkinson’s disease.

Due to factors such as the loss of dopaminergic neurons in the substantia nigra, abnormalities in the basal ganglia circuit, pathological aggregation of alpha-synuclein, and neuroinflammation, PD patients often experience a variety of motor symptoms (Balestrino and Schapira, 2020). Bradykinesia is the core motor symptom and is defined as a decrease in the speed, amplitude, and flexibility of voluntary movement (Xu et al., 2025). Specific manifestations include difficulty starting, decreased speed (hypokinesia), amplitude reduction, impaired rhythm and coordination, and prolonged duration of movement (Bologna et al., 2016). Affects facial expression (Bologna et al., 2013), writing and other fine hand movements (Matejicka et al., 2024), speech status, and expression (Cavallieri et al., 2023) in activities of daily living. Tremors are generally categorized into static tremors, postural tremors, and action tremors (Abusrair et al., 2022). Among these, static tremor is the hallmark symptom of PD, characterized by regular, rhythmic oscillations that occur when the patient is at rest, such as while sitting or standing (Gironell et al., 2018). This type of tremor commonly begins in the hands, most commonly affecting the index finger and thumb, but it can also involve the chin and mouth (Fabbri et al., 2017). Patients may also experience postural tremors, which occur due to muscle tension while maintaining specific postures. These tremors primarily affect the limbs and head, especially when the patient maintains an upright posture or extends their arms (Dirkx and Bologna, 2022). In addition, tremors related to movements are also quite common, presenting with a slightly higher frequency, typically ranging from 4 to 8 Hz, and smaller amplitudes (Wenzelburger et al., 2000). This type of tremor often occurs at the beginning of the movement and may progressively intensify with continuing, particularly during fine activities such as writing or grasping objects (Kraus et al., 2006). Rigidity is characterized by increased involuntary tension in limb muscles that cannot be fully relaxed even at rest. The patient’s limbs exhibit sustained resistance during passive activity, similar to bending a “lead pipe” (Guayacán et al., 2025). As the examiner slowly and passively bends or extends the patient’s limbs, an intermittent, cogwheel-like resistance is felt (Ghiglione et al., 2005). Due to the continuous increase in muscle tone, patients may hold on to a flexed posture, which includes a forward tilt of the head, forward flexion of the trunk, and slight flexion of the elbows and knees (Xu et al., 2025). Therefore, patients often feel pain or muscle fatigue, which affects their daily living abilities such as walking, writing, dressing, and fine motor skills (Kumar et al., 2025). Figure 1 shows the motor symptoms and corresponding characteristic manifestations of PD.

## 3 AI and its role in auxiliary diagnosis

**FIGURE 2:**
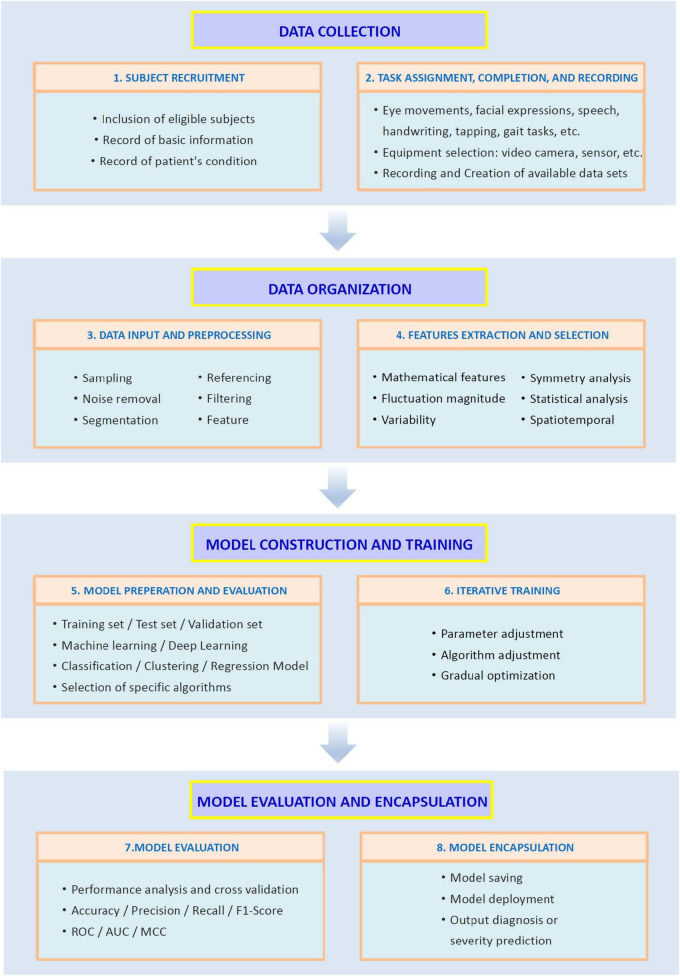
The application process of artificial intelligence technology in Parkinson’s disease adjunctive diagnosis.

Machine learning is a subfield of AI that enables computer systems to automatically learn and make predictions or decisions through data-driven approaches (Sarker, 2021a). In recent years, for instance, ML-based methods have demonstrated promising outcomes in medical diagnosis research (Izonin et al., 2023). DL is a specialized area within ML that utilizes artificial neural networks, particularly deep neural networks, to simulate the functioning of the human brain (Sarker, 2021b). DL has the ability to automatically learn intricate patterns within data and shows excellent performance when dealing with large-scale data, making it particularly suitable for tasks like natural language processing, image or speech recognition (Deng, 2015; Su and Li, 2020). In medical diagnosis ML techniques are effectively employed to identify diseases based on datasets derived from patient symptoms. In this process, doctors play a crucial role in augmenting or validating the decision-making of AI to ensure appropriate decisions about diseases with reasonable accuracy (Richens et al., 2020). Over the past few years, ML algorithms have assisted in resolving more complex problems and have emerged as valuable supplementary tools for doctors (Gagliano et al., 2017; Zhou et al., 2019). By uncovering hidden information in data that was previously overlooked in PD clinical diagnosis (Kononenko, 2001), these algorithms have significantly enhanced diagnostic accuracy in the healthcare industry. Due to the exponential changes brought about by the digital revolution, the collection, utilization, storage, and sharing of medical data have become a reality. The progress of technology has spurred the increasing sophistication of algorithms, enabling more accurate and efficient data applications. As a result, AI technology is playing an even greater role in medical diagnosis. Based on this, we summarized the clinical manifestations of PD motor symptoms and their application in auxiliary diagnosis. Figure 2 shows the application process of artificial intelligence technology in PD adjunctive diagnosis.

## 4 Eye movement

The eye movement functions of patients with PD exhibit significant abnormalities, primarily affecting fundamental tasks such as saccades, fixation, and smooth pursuit. Saccadic movement in PD patients is usually slow and uncoordinated, with a prolonged scanning latency, reduced velocity, and decreased movement amplitude compared to healthy individuals (Lohnes and Earhart, 2011). In short-distance saccade tasks, both the accuracy and speed of eye movements are notably impaired, which is closely associated with dysfunction in the basal ganglia (Jung and Kim, 2019; Armstrong, 2015). Furthermore, PD patients often experience frequent gaze instability, manifesting as nystagmus and micro-saccades. These disturbances, with an average fundamental frequency of 5.7 Hz, disrupt the stability of visual fixation on targets (Gitchel et al., 2012). Deficits in smooth pursuit are also pronounced in PD patients, particularly during low-frequency tracking tasks. Patients frequently struggle to maintain smooth tracking of moving targets and exhibit frequent saccadic intrusions (Li et al., 2023). To elicit these abnormal movements, clinicians commonly use a pen or something with a point for the patient to focus on, then move the point in various directions. These tasks typically involve holding the object still and asking the patient to maintain fixation (fixation) or moving the object back and forth or up and down (smooth pursuit). Physicians observe abnormalities in patients’ eye movements as they perform tasks. However, subtle movements such as nystagmus can be missed by the naked eye, even by experienced clinicians. These eye movement abnormalities may serve as valuable biological markers for early diagnosis and clinical assessment of PD.

With the advent of AI, capturing these delicate abnormal eye movements through video and analyzing them with software has become feasible. Usually, various eye movement tasks induce pupil responses and are then recorded as videos. The first step in video analysis involves pupils detecting, boundary refining, and accurately locating (Kassner et al., 2014). Given the subtle nature of abnormal eye movements in PD patients, it is essential to employ visualization techniques that magnify the eye movements and filter out interferences, such as those caused by head tremors. Techniques like virtual overlay of the pupil center and contour ellipse, background subtraction (Bustos et al., 2023), and Eulerian Video Magnification (EVM) are commonly used to enhance the visibility of the movements (Wu et al., 2012; Williams et al., 2020a). After processing, algorithms are applied to track and quantify the eye movements, thus providing objective data on abnormalities. Finally, based on the extracted objective data, a recognition model is constructed to achieve the identification and detection of PD. For instance, Brien et al. (2023) gathered video-recorded eye-tracking measurements from 104 PD patients and 106 healthy individuals participating in an alternating pro/anti-saccade protocol. By analyzing features such as saccades, pupil behavior, and blink patterns, they trained an ML classifier to predict confidence scores for diagnoses. Using a Linear Mixed Model, they determined disease probabilities based on eye-tracking biomarkers. The classifier achieved a sensitivity of 0.83, a specificity of 0.78, and a Receiver Operating Characteristic Area Under the Curve (ROC-AUC) of 0.88. The predicted confidence scores correlated with motor and cognitive performance in PD patients, demonstrating that eye-tracking markers can reflect these clinical scores dependably. This finding highlights the potential of eye-tracking technology as a non-invasive method for prodromal screening of PD. Similarly, Bejani et al. (2022) employed the same technology to analyze smooth tracking eye movements. They measured various parameters, including complexity based on entropy and regularity, which describe the dynamic characteristics of the system and assess self-similarity. Using a Support Vector Machine (SVM) for classification, they achieved an accuracy of 0.74 (sensitivity: 0.73, specificity: 0.74) in PD diagnosis. This further supports the utility of eye-tracking data, particularly the dynamic system features, in differentiating PD patients from healthy subjects.

Eye tracking also shows great potential in the clinical and social application of PD. A study validated the use of consumer-grade eye-tracking systems like Eye Tribe to detect PD-relevant biomarkers with comparable accuracy to clinical-grade devices, demonstrating the feasibility of accessible and cost-effective eye-tracking tools in real-world settings (Szymański et al., 2017). Virtual reality (VR) environments integrated with machine learning have also shown potential. A study using VR-based eye-tracking and SVM algorithms achieved high classification performance between PD and control groups, highlighting the benefits of immersive, standardized testing platforms for inducing and measuring disease-relevant visual behaviors (Jiang et al., 2024). Moreover, deep transcranial magnetic stimulation studies have linked eye-tracking metrics (e.g., saccade rate, fixation duration) to neuropsychological assessments, suggesting that eye movement parameters could serve as sensitive markers not only for motor but also for cognitive symptom monitoring (Cont et al., 2025). Beyond diagnosis, machine learning models have also been used to predict disease severity based on eye movement data, aiding in personalized treatment planning and progression tracking (Chudzik et al., 2020). Beyond conventional eye-tracking, mixed reality environments offer innovative ways to record eye-gaze behaviors under naturalistic conditions. A study proposed an MR-based system to assess eye movements in PD patients, demonstrating that wearable and wireless MR glasses could facilitate home-based, real-time diagnosis, providing a scalable and user-friendly solution for continuous monitoring (Daniol et al., 2024). In terms of technical innovation, researchers are also exploring Human-in-the-Loop systems, where clinician gaze data improves ML model selection of diagnosis-relevant features in eye images. This hybrid approach enhances both accuracy and interpretability, highlighting the synergy between human expertise and algorithmic efficiency (Fong et al., 2016). In recent years, the availability of wearable sensors has been increasing, and the ability of machine learning algorithms to process multimodal and high-frequency gaze data has been enhanced (Zhao et al., 2024). With the increasing popularity of eye-tracking data as a digital biomarker, it is conducive to promoting early detection and personalized disease tracking (Sekar et al., 2024).

While ML-based eye movement tracking technology has made notable improvement in recognizing PD, there are still several challenges that need to be addressed. Head movements, especially head tremors, can interfere with eye movement tracking, making it difficult to differentiate between vestibular reflexes and the eye response characteristics of the disease (Gitchel et al., 2012). Although some studies have attempted to reduce this impact by isolating head tracking from eye tracking (similar to a 2D user interface element) (Orlosky et al., 2017), further technological advancements are needed to reduce the impact of head tremors on eye movement analysis. Additionally, using VR glasses and similar devices to record eye movements may cause adverse reactions such as dizziness in some patients (Hu et al., 2023), highlighting the need for new, more comfortable devices for eye movement tracking. Many elderly patients also suffer from presbyopia or other refractive errors (Loh and Ogle, 2004), which can affect the accuracy of visual tracking tasks. Moreover, large-scale studies are still needed to widely validate the automatic prediction capabilities of this technology. Furthermore, differences in pupil dilation during eye movement tracking remain a topic for future investigation, as this phenomenon may also have diagnostic significance (Orlosky et al., 2017).

## 5 Facial expression

A hallmark feature of PD is “hypomimia,” characterized by the reduction or absence of facial expressions (Goetz, 2011). Patients typically exhibit a decreased blinking rate and a rigid, unnatural facial appearance, even during voluntary attempts to express emotions (Wu et al., 2014). Facial muscle dysfunction contributes to this phenomenon, leading to a significant reduction in the intensity and duration of spontaneous expressions. Additionally, the ability to mimic facial expressions, particularly those associated with emotions like happiness or anger, is severely impaired (Kang et al., 2019). Moreover, patients show a pronounced delay in the speed of emotional responses (Bowers et al., 2006). Upper facial dyskinesia is most evident in the reduced frequency of spontaneous blinking and prolonged pauses between eyelid closure and reopening (Agostino et al., 2008). Lower facial motor abnormalities are primarily reflected in expressive movements such as smiling. For instance, the peak velocity and amplitude of lip corner movements during postural or voluntary smiles are notably reduced, with these kinematic deficits strongly correlating with the severity of bradykinesia in the limbs (Marsili et al., 2014). Furthermore, patients frequently struggle to initiate expressions that align with their actual emotional states (Simons et al., 2004). In contrast to the typically asymmetric motor symptoms of PD, facial expression deficits are generally bilaterally symmetrical (Bologna et al., 2013). However, isolated cases of asymmetrical facial hypoexpression have been documented (Phillips et al., 2020; Panichelli and Spitz, 2021; Kurtis et al., 2019). Studies employing facial electromyography and action unit analysis have revealed significantly reduced muscle activity in the periocular region and at the corners of the mouth in PD patients compared to healthy controls (Priebe et al., 2015; Smith et al., 1996). These muscular deficits further exacerbate limitations in expressive capacity. The severity of facial expression impairment in PD varies widely. While mild cases may present with a slightly dull expression, severe cases can result in a complete loss of facial expressivity (Gunnery et al., 2017). This deficit not only hampers emotional expression and social interaction but also serves as a valuable clinical indicator for assessing the severity of motor dysfunction in PD.

The implementation of facial recognition for PD using AI involves several key steps. The first step is to record the subject’s facial expressions under appropriate lighting conditions, typically using video or photographs. These recordings are then analyzed frame-by-frame, followed by standardized processing of the facial images (Jabberi et al., 2023), such as alignment using the OpenCV library. In terms of ML, facial key features, such as texture characteristics and facial landmarks, need to be extracted (Krithika and Priya, 2021). Finally, an AI recognition model is constructed for facial identification, which is optimized through iterative training to enhance accuracy. Image-based facial expression recognition is typically 60%–90% accurate (Grammatikopoulou et al., 2019; Rajnoha et al., 2018), while frame-by-frame video analysis can achieve 85%–95% accuracy (Bandini et al., 2017; Gómez-Gómez et al., 2022; Hou et al., 2021; Hou et al., 2022; Jin et al., 2020). With the advancement of AI technology, the generative neural network model on a small dataset has even achieved a 100% recognition rate (Huang et al., 2024).

Facial analysis technologies have demonstrated considerable potential in diagnosing PD by detecting micro-expressions, facial asymmetry, and reduced action unit activation. Action Unit detection and transfer learning are central to many studies, such as the Facial Region Awareness framework, which improved the consistency of facial region representation across tasks, enhancing downstream classification performance (Gao and Patras, 2024). For instance, Ali et al. (2021) developed a facial micro-expression classifier achieving 95.6% accuracy using Support Vector Machines, identifying lower variance in action units (e.g., AU6, AU12, AU4) in PD patients compared to controls. Similarly, Gómez-Gómez et al. (2022) used facial expression sequences with affective-domain adaptation and deep learning to model hypomimia, improving detection accuracy up to 87.3%. Emerging multimodal models have been particularly effective. For example, Kyprakis et al. used 3D CNN-LSTM and Swin Transformer models on facial video data to predict depressive symptoms–a non-motor symptom of PD–with over 94% accuracy, providing a non-invasive method for comprehensive assessment (Kyprakis et al., 2025). Adnan et al. (2023) proposed an AI framework using smile videos from over 1,000 participants to distinguish PD patients with over 87% accuracy and noted generalizability across diverse populations. Privacy and accessibility have also been addressed. Jiang et al. (2022) proposed a privacy-preserving AIoT edge framework using encrypted facial data to monitor PD patients undergoing deep brain stimulation (DBS), showing facial features could reflect treatment efficacy. Due to the limited dataset, enhancing the training accuracy and generalization capacity of the model poses a significant challenge. Therefore, Huang et al. (2024) used StarGAN to synthesize facial expressions of PD patients and combined these with a Swin Transformer to integrate multi-modal features. Their model achieved 100% diagnostic accuracy on data from 95 PD patients. A particularly notable direction is the use of self-administered or home-based diagnostic tools. Mishra, for example, developed an application based on the Facial Action Coding System and neural networks, allowing users to assess Parkinson’s symptoms remotely (Mishra, 2021). Facial expression analysis, powered by deep learning and computer vision, is emerging as a powerful and non-invasive biomarker for PD diagnosis and monitoring. These systems, especially when combined with privacy-aware frameworks and large-scale video datasets, show promise in supporting clinicians and enabling remote health assessments.

However, there are still several issues that warrant attention. AI models rely heavily on large, high-quality datasets for training and validation. However, existing PD facial recognition datasets are generally small and lack sufficient diversity to capture all possible facial variations. PD patients exhibit significant individual differences in facial expression changes (Xu et al., 2022), which are closely related to the progression of the disease, individual characteristics (e.g., gender, age, disease duration), and treatment approaches (e.g., medication or deep brain stimulation) (Palmeri et al., 2020). AI models may have limitations when addressing these individual differences, potentially affecting their accuracy and generalizability. This issue is particularly pronounced in early-stage patients, where facial feature changes are minor. Currently, there is a lack of targeted research on early-stage PD patients, and traditional AI techniques may struggle to capture sufficient features. Additionally, PD patients often experience comorbid emotional disorders such as depression or anxiety, which significantly impact their facial expressions. These emotional disorders, combined with the disease’s characteristic “masked face,” complicate the accurate expression of emotions (Jin et al., 2017). Consequently, AI models may struggle to accurately capture the patient’s emotional state, leading to incorrect emotion recognition. Furthermore, the expression of emotions is influenced by social, cultural, contextual, and individual differences, all of which increase the difficulty of emotion recognition.

## 6 Speech features

**FIGURE 3:**
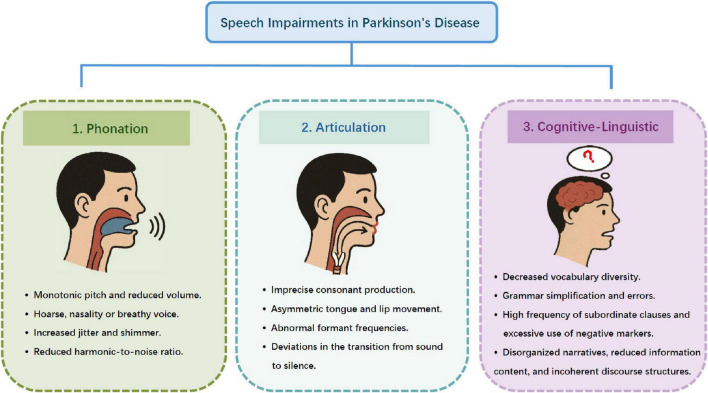
Speech impairment in Parkinson’s disease.

Pathological changes in PD significantly affect the control of speech muscles, drawing widespread attention to the speech characteristics associated with the condition. Patients often exhibit abnormalities across multiple dimensions, including intonation, articulation, fluency, and rhythm of language expression, which serve as key diagnostic criteria (Karan et al., 2020a; Azadi et al., 2021). In the early stages of PD, monotonic pitch and volume are common, which may gradually progress into hypophonia (Jankovic, 2008; Kursun et al., 2012). Increased breathiness, hoarseness, and nasality are particularly noticeable during phonation in the early to mid-stage PD. These symptoms are likely attributed to laryngeal rigidity or bradykinesia (Shahbakhti et al., 2013). Compared with healthy individuals, PD patients show significant alterations such as increased frequency jitter and intensity shimmer, alongside a reduced harmonic-to-noise ratio (Azadi et al., 2021). These findings suggest incomplete vocal cord closure and weakened motor function of the vocal cords, especially in individuals with advanced disease (Rusz et al., 2015; Holmes et al., 2000). Acoustic analyses further reveal imprecise consonant production, asymmetric articulation, and abnormal formant frequency distributions, underscoring the pronounced motor control deficits in the vocal organs caused by PD (Arias-Vergara et al., 2017; Mawdsley and Gamsu, 1971). Dynamic analyses highlight significant deviations in the transition from sound to silence, including delays in onset and offset (Laganas et al., 2022). Additionally, irregular speech rates, unwarranted pauses, and disruptions in speech fluency reflect the impact of bradykinesia on pronunciation control (Arias-Vergara et al., 2017; Holmes et al., 2000; Laganas et al., 2022). The severity and manifestation of these speech characteristics vary with disease progression. While early-stage patients exhibit mild speech suppression, late-stage patients are more likely to experience unclear articulation, impaired respiratory control, and vocal cord lesions (Kovac et al., 2021; Shahbakhti et al., 2013). In addition to vocal impairments, patients with PD often exhibit significant linguistic abnormalities. In terms of morphology, unsupervised morphological segmentation reveals abnormal distributions of morpheme categories (prefixes, stems, suffixes) (Eyigoz et al., 2018). Cross-linguistic studies conducted in Spanish, German, and Czech have identified specific verbal markers. PD patients display distinct morphological patterns, lexically, there is a noticeable decline in lexical diversity. Patients frequently repeat words and use fewer common and proper nouns (Yokoi et al., 2023). While the total number of verbs may increase, there is a marked reduction in the use of action verbs (e.g., “play,” “take”) (Escobar-Grisales et al., 2023a). Semantically, patients have deficits in processing action verbs (e.g., “run,” “jump”) (García et al., 2022). Latent semantic analysis shows weaker associations between these verbs and action concepts, and patients rely more on non-action domains (e.g., “read,” “say”) (García et al., 2016). Grammatically, patients tend to produce syntactically simplified utterances, characterized by shorter sentence lengths, reduced dependency distances, and fewer noun, verb, and prepositional phrases. The frequent use of repetitive fillers (e.g., the Spanish filler “pues”) is also observed (Pérez-Toro et al., 2019). Additionally, PD patients exhibit an increased rate of grammatical errors and a higher frequency of subordinate clauses (e.g., those beginning with “because” or “although”) (García et al., 2016). They also tend to overuse negation markers, produce fewer segmented sentences, and rely more heavily on digressive syntactic constructions (García et al., 2016). Sometimes, a skewed distribution of verb tenses (e.g., a preference for the present tense), and inconsistent case/gender marking in determiners and pronouns can also occur (Eyigoz et al., 2020). At the discourse level, impairments include disorganized narratives, a decrease in informational content (fewer correct units in picture descriptions), and incoherent structures (e.g., overused digressions, fewer full stops) (Favaro et al., 2023b). In addition, patients also exhibit impairments in syntactic processing and emotional language processing. Joint assessment of grammatical comprehension and social emotional processing can serve as a multidimensional linguistic marker for early diagnosis (Baez et al., 2020). The summary of the three aspects of speech characteristics in PD is shown in Figure 3.

Speech analysis encompasses several key aspects, including phonation, articulation, prosody, and cognitive-linguistic elements. Phonation refers to the physiological processes involved in speech production, primarily focusing on the vibration of the vocal cords and the generation of sound. This process includes the initiation, maintenance, and cessation of sound, as well as the regulation of pitch and volume (Dromey et al., 1995). Articulation involves the movement and positioning of the articulatory organs, particularly the mouth, tongue, lips, and other related structures. Articulation ensures speech clarity and accuracy, enabling sounds to be effectively transformed into intelligible language (Kuruvilla-Dugdale et al., 2020). Prosody primarily examines paralinguistic features, such as variations in pitch, syllable rate, and the expression of emotions within the speech signal (Beier et al., 2025). Cognitive-linguistic approaches, on the other hand, focus on analyzing cognitive behavior deviations by assessing factors such as vocabulary usage, sentence complexity, phrase construction, and the occurrence of word repetitions, among other indicators (Miller, 2017). The process of classifying speech recognition with the assistance of AI involves several key steps. Initially, a pronunciation task is assigned, typically comprising long vowels and standard sentences, followed by the collection of high-quality audio recordings using a high-definition recorder (Asci et al., 2020). Next, the recorded speech samples undergo preprocessing, which includes speech signal classification and filtering techniques to remove potential noise. Subsequently, relevant speech features are extracted, and a recognition model is developed (Eyben et al., 2013). Finally, the constructed model is tested to evaluate its performance.

Parkinson’s disease-related speech datasets are designed to help diagnose and assess the symptoms and progression of PD by collecting articulatory features from patients. The currently commonly used datasets listed in Table 1, which contain different numbers of patients and healthy controls, cover various languages (e.g., Spanish, Italian, Czech, English, etc.) and various speech tasks. For example, the PD Classification dataset (Sakar et al., 2018) from the University of Istanbul classifies patients using repeated vowel articulations, while the Parkinson’s Telemonitoring dataset (Tsanas et al., 2010) uses 16 phonological features to predict a patient’s motor and global UPDRS scores. The PC-GITA (Orozco-Arroyave et al., 2014) and Neurovoz datasets (Mendes-Laureano et al., 2014) provide rich articulatory tasks to analyze articulatory dynamics and dysarthria in native Spanish-speaking patients. The Johns Hopkins Medicine dataset (Favaro et al., 2023a) focuses on spontaneous speech and read-aloud tasks in English to investigate the relationship between language fluency and PD. The Italian PVS dataset in Italy focuses on vowel articulation and read-aloud tasks in Italian (Dimauro et al., 2017). The dataset from the UC Irvine ML Repository also comprises a series of voice recordings of PD patients (Carlos, 2016; Hlavnika et al., 2017; Little, 2007; Olcay et al., 2013; Sakar et al., 2018; Tsanas et al., 2010).

**TABLE 1:** Summary of the publicly available speech datasets related to Parkinson’s disease.

Dataset name	Number of participants	Task(s) involved	Cite
Parkinson’s disease classification (Istanbul)	188 PD, 64 HC	Sustained pronunciation of three repeated vowels (/a/)	Sakar et al., 2018
Parkinson’s telemonitoring	42 PD	Sustained vowel phonations ranging from one to 36 s in length	Tsanas et al., 2010
PC-GITA	50 PD, 50 HC	Continuous pronunciation, 45 words, 10 sentences, 1 reading text, 1 monologue task in Spanish	Orozco-Arroyave et al., 2014
Neurovoz	54 PD, 58 HC	Continuous vowel pronunciation, DDK testing, 16 structured listening, repeated persuasion, monologues in Spanish	Mendes-Laureano et al., 2014
Johns Hopkins medicine (JHM) dataset	23 PD, 27 HC	Spontaneous speech task for image description, two short text reading tasks in English	Favaro et al., 2023a
Italian PVS	22 elderly HC, 28 young HC, 28 PD	Balanced pronunciation of 5 long vowels, reading short texts, phrases, and words in Italian	Dimauro et al., 2017
Czech PD	20 PD, 15 HC	Sustained phonation, sentence repetition, passage reading, monologues in Czech	Dimauro et al., 2017

Research on AI-driven speech recognition for PD has reached a relatively mature stage, with significant advancements in methodologies ranging from traditional ML to DL. A key transition has been made from manual extraction of voiceprint features to fully automated extraction processes (Escobar-Grisales et al., 2023b). Current studies primarily focus on DL techniques, including deep acoustic feature extraction (DAFE) (Khojasteh et al., 2018), end-to-end (E2E) learning (Quan et al., 2022; Rios-Urrego et al., 2022), and transfer learning (TL) (Reddy et al., 2024). Among these, convolutional neural networks (CNNs) (Er et al., 2021) are widely utilized in E2E models, while Transformer-based architectures are gaining increasing popularity (Malekroodi et al., 2024). DAFE enhances the interpretability of results by examining how deep phonetic features influence ML and other DL methods. The recognition accuracy of the DAFE method is usually above 85% (Favaro et al., 2023b; Karan et al., 2020b; Mallela et al., 2020b). Wav2Vec2.0, VGGish, and Soundnet extraction of deep acoustic features (Ferrante and Scotti, 2023) as well as autoencoders combined with deep bilateral learning (Ma et al., 2021) can achieve more than 90% accuracy and even up to 99.6%. E2E and TL typically have better presentation performance (van Gelderen and Tejedor-García, 2024). E2E models, especially those using the Transformer architecture, require relatively larger clinical datasets and computational resources, with more applications currently (Hemmerling et al., 2023). It typically achieves more than 95% accuracy (Hireš et al., 2022; Hireš et al., 2023; Mallela et al., 2020a; Reddy et al., 2024), and even 100% in models combining CNNs and LSTMs (Boualoulou et al., 2023). In contrast, TL enables the transfer of pre-trained models across various linguistic datasets, reducing computational costs while improving multilingual adaptability (Hireš et al., 2023; Vásquez-Correa et al., 2021).

Speech features have been widely used in the diagnosis of PD for example, a study introduced the PPINtonus system, which integrates tonal analysis with a GAN-augmented deep neural network trained on phonetic data collected under real-world conditions. Utilizing PRAAT software to extract acoustic features, such as Mel-Frequency Cepstral Coefficients and Shimmer, from 120-s voice samples, the system achieved an impressive classification accuracy of 92.5% (Reddy, 2024). García-Ordás et al. (2024) proposed a multitask multilayer perceptron model capable of performing both classification and regression tasks based on voice data. Their approach attained over 99% accuracy in distinguishing between severe and non-severe PD cases by leveraging features such as jitter, harmonic-to-noise ratio, and articulation rate. Verbal biomarkers such as increased pause duration, word retrieval delays, and reduced speech fluency are commonly assessed using both theory-driven psycholinguistic models and data-driven embedding techniques. For instance, Padhee et al. (2020) showed that these features can effectively distinguish PD from other cognitive disorders with high diagnostic accuracy. Mir et al. (2024) developed an LSTM-based framework, demonstrating that the temporal dynamics of speech and movement can be informative for diagnosis, even in the absence of overt motor symptoms. However, its recognition accuracy is usually between 70 and 90% (Karaman et al., 2021; Vásquez-Correa et al., 2021). Despite these technological breakthroughs, most existing studies rely on voiceprint features extracted from limited public datasets. These datasets vary in terms of geographical and linguistic diversity, as well as in the content of the original audio recordings. Consequently, a standardized approach for collecting acoustic features has not been established. Further research is required to develop standardized voiceprint acquisition protocols and to explore PD speech characteristics across different ethnic subgroups within various language regions.

## 7 Handwriting and tapping

Handwriting and tapping tasks serve as valuable means for assessing the fine motor skills of the upper limbs in PD patients. In handwriting, PD patients often demonstrate progressively smaller writing (micrographia), along with irregular character shapes and reduced legibility (Letanneux et al., 2014). Due to bradykinesia and tremor, maintaining consistent stroke speed and pressure becomes challenging, leading to slower writing and non-standardized strokes (Zham et al., 2019). In finger-tapping tests, PD patients typically show a marked reduction in tapping speed, diminished amplitude, and difficulty maintaining rhythmic consistency, attributed to bradykinesia and muscle rigidity (Giovannoni et al., 1999). This impairment is particularly evident during complex tasks requiring synchronized multi-finger movements or alternating repetitive tapping, where movement accuracy and coordination decline markedly (Agostino et al., 1998). In more intricate hand tasks, such as coin rotation or manipulating small objects, PD patients often exhibit reduced independence of finger movements, a condition known as limb-kinetic apraxia (Syeda et al., 2022). As the frequency of hand movements increases, their amplitude and coordination deteriorate further, highlighting movement frequency as a critical factor influencing motor performance in PD patients (Stamatakis et al., 2010).

The most commonly used devices for diagnosing PD through handwriting analysis are drawing tablets (Jackson, 2015) and/or biometric smart pens (Bashir and Kempf, 2012). These devices typically provide parameters such as the X and Y coordinates of the trajectories drawn by the subject on a tablet or in the air, sampling time, pen orientation angles (azimuth and elevation), and the pressure applied by the pen during writing. With these devices, subjects are asked to perform simple drawing tasks (e.g., spirals, meandering shapes, and circles), basic writing tasks (such as writing one or more cursive letters, or continuous and repeated letters like “lll” or “lele”), as well as more complex writing tasks (e.g., copying detailed, intricate text) (Aouraghe et al., 2023). The collected data is first preprocessed by filtering to reduce noise and smoothing the signals (e.g., normalizing signal duration) (Impedovo and Pirlo, 2008). Subsequently, relevant kinematic features, mechanical properties, spatiotemporal features, entropy, and energy characteristics are extracted (Afonso et al., 2019; Drotár et al., 2015; Drotár et al., 2016), and finally, recognition models are built based on the data types. There are currently several PD handwriting or drawing datasets available to the public (Table 2). Those contain handwriting samples from PD patients and healthy individuals, with task types including Archimedean spiral, repeated letters and words, drawing circles, winding lines, and static and dynamic spirals. These tasks aim to detect changes in fine motor control, stability, and fluency in PD patients to assess their motor coordination and handwriting ability. Multiple datasets provide valuable resources for early diagnostic tools and monitoring models by comparing differences in writing behavior between healthy individuals and PD patients.

**TABLE 2:** Summary of the publicly available handwriting or drawing datasets related to Parkinson’s disease.

Dataset name	Number of participants	Task(s) involved	Cite
Parkinson’s disease handwriting (PaHaW)	37 PD, 37 HC	Archimedean spirals, repetitive loops, the letter “l,” syllable “le,” Czech words, Czech sentence	Drotár et al., 2016
PD multi MC	16 PD, 16 HC	Repetitive longitudinal letters, triangular waves, rectangular waves, repetitive English words “Monday” and “Tuesday,” repetitive subject names	Taleb et al., 2017
Hand PD	74 PD, 18 HC	Spiral drawing	Pereira, 2021
New hand PD	31 PD, 35 HC	Circles, spiral, meander, and signals handwriting	Xu and Pan, 2020
Parkinson disease spiral drawings using digitized graphics tablet	62 PD, 15 HC	Spirals and stability test on certain point	Isenkul and Betul, 2017

Before 2020, the recognition accuracy based on handwriting in ML and DL typically ranged from 80 to 90% (Cascarano et al., 2019; Drotár et al., 2016; Gupta et al., 2018; Kotsavasiloglou et al., 2017; Mucha et al., 2018; Mucha et al., 2019; Xu and Pan, 2020; Zham et al., 2018). However, since 2022, due to advancements in neural networks, ML has seen significant improvements. Specifically, KNN, XGBOOST, and ChiGa-Net models can now achieve an accuracy rate of 92%–99% (Abdullah et al., 2023; Diaz et al., 2019; Shrivastava et al., 2024). Meanwhile, in DL, multiple deep CNN models and deep transfer learning have achieved an accuracy rate exceeding 98% (Kamran et al., 2021; Naz et al., 2023; Pereira et al., 2016). Remarkably, in RNN-LSTM and RNN-BLSTM, as well as VGG16 and VGG19, the accuracy has reached 100% (Kumar and Ghosh, 2024; Zemmar et al., 2025). Studies have shown that AI can effectively extract features from dynamic handwriting signals (e.g., pen pressure, speed, acceleration, stroke patterns) to distinguish between healthy individuals and PD patients. Models like Convolutional Autoencoders (CAE), Transformers, Recurrent Neural Networks, and Multilayer Perceptrons (MLPs), are commonly used to analyze these spatiotemporal data modalities, demonstrating high classification accuracy ranging from 85% to over 95% in certain datasets (Aldujaili et al., 2025). For instance, Kansizoglou et al. (2025) proposed a hierarchical DL model to detect PD from online handwriting, focusing on drawing-aware features. Kasab et al. (2024) demonstrated how CAE can classify spiral and wave drawings with high sensitivity, while Arasavali et al. (2024) used an MLP-based framework to dynamically assess handwriting traits for PD classification.

The use of GAN-augmented datasets and transformer-based models further enhances the generalization and robustness of PD classifiers, particularly in early detection stages when symptoms are subtle (Darkhal and Sedaghat, 2025). Saha et al. (2024) leveraged multimodal handwriting signals, including micrographia, spiral, and meander patterns, integrated with ML for high-precision diagnosis. Kamireddy et al. also emphasized the value of such non-invasive digital biomarkers through spiral and wave test analysis using deep learning pipelines (Kamireddy et al., 2025). Notably, these models are often integrated into portable digital platforms, such as tablets or smart pens, enabling remote and cost-effective PD screening. This democratizes access to neurological assessments, especially in underserved regions or for elderly populations unable to access specialized centers. Its integration into telemedicine tools could revolutionize how we screen and monitor neurodegenerative diseases. However, the majority of these studies rely on existing datasets. Even in the widely recognized largest PD handwriting dataset, the number of patients is limited to just 74. Additionally, most of the populations studied are from countries with alphabetic languages. Consequently, these subjects may perform better in tasks such as drawing curves, circles, and waves compared to populations using stroke-based languages like Chinese. Therefore, it is essential to expand the sample size and include individuals from diverse linguistic backgrounds. Furthermore, cognitive impairment levels vary significantly among patients, leading to considerable differences in drawing performance. For instance, some patients may struggle to complete handwriting or drawing tasks due to inattention or memory loss. Future research must focus on increasing both the size and diversity of datasets by collecting handwriting and drawing data from various populations and disease stages, thereby improving the generalizability of AI models. Additionally, it is crucial to account for emotional and cognitive factors. Specifically, when analyzing handwriting and drawing data, a comprehensive consideration of the patient’s emotional and cognitive state is necessary to enhance diagnostic accuracy. Furthermore, the development of user-friendly, low-cost devices for data collection, along with the establishment of standardized protocols and operating procedures, will be critical in facilitating the clinical application of these technologies.

For finger-tapping, some existing work suggests the use of wearable sensors (Pasluosta et al., 2015). A recognition model was built using parameters such as speed and acceleration of the tapping, and the accuracy of the X-ception and SVM models was 71.3% (Singh et al., 2023) and 87.0% (Shin et al., 2024), respectively. However, these sensors inevitably come into physical contact with PD patients, which may affect the patient’s movement due to the weight and contact of the sensors. In addition, the data collected by the sensors are affected by gravity, thus complex procedures are required for calibration (Shima et al., 2019). Some studies use colored markers or gloves (Buongiorno et al., 2019; Krupicka et al., 2017) to facilitate visual capture, catch subtle movements by using video cameras, especially the depth camera in recent years (House et al., 2017), and measure the relative direction and pixel-level distances of finger movements (Khan et al., 2014; Liu et al., 2019; Williams et al., 2020b). It is capable of detecting and estimating hand joint positions and postures, thereby extracting 3D hand kinematic features. Notably, 2D or 3D vision can not only achieve an accuracy of over 85% in binary models (identifying PD and healthy individuals) (Amprimo et al., 2023; Buongiorno et al., 2019; Kajan et al., 2024; Khan et al., 2014; Monje et al., 2021), but also evaluate the classification of patients’ movement disorders in multi-class models, but the accuracy is usually lower than that of binary models, between 70 and 90% (Guarín et al., 2024; Guo et al., 2022; Li et al., 2021; Li et al., 2022; Park et al., 2021; Yang et al., 2022; Yu et al., 2023). The use of smartphone applications (Lee et al., 2016; Surangsrirat et al., 2022) for self-management of PD assessment is becoming increasingly popular. For instance, participants were instructed to use their index finger to rapidly and repeatedly click on both sides of a rectangle displayed on the touchscreen. Subsequently, indicators such as click accuracy and movement distance are used for disease assessment (Lee et al., 2016). PDGs is one such application that has achieved over 94.5% recognition accuracy on various smartphones (Teng et al., 2023). It is noteworthy that one system employed a k-nearest neighbor classifier to rapidly facilitate the differential diagnosis of repetitive finger-tapping features captured by the gyroscope within a wearable system equipped with inertial sensors (Belić et al., 2023). This system can effectively distinguish among patients with PD, those with atypical Parkinson’s syndromes such as progressive supranuclear palsy and multiple system atrophy, and healthy controls (HC). In multi-classification tasks, it achieved an overall classification accuracy of 85.18%.

## 8 Gait

Gait abnormalities in PD are crucial indicators for diagnosis and monitoring, reflecting multiple impairments in the patient’s motor system. Dynamic feature analysis shows that PD patients exhibit significant decreases in stride length, step duration, stance time, swing time, and support time, with shortened stride length, reduced walking speed, and decreased gait rhythm being particularly substantial (Mirelman et al., 2019; Wahid et al., 2015; Wang et al., 2016; Zhao et al., 2021). It is worth noting that although gait rhythmicity is reduced, PD patients can improve gait to some extent by adjusting step frequency under external prompts, which provides a new direction for gait treatment (Sarbaz et al., 2012; Wu and Krishnan, 2010). Regarding spatial characteristics, PD patients tend toward reduced forward displacement and increased vertical displacement when walking forward, with these changes becoming more pronounced as the disease progresses (Hausdorff, 2009; Ogata et al., 2022). Furthermore, gait freezing (FoG) is a hallmark symptom of PD, characterized by a sudden cessation of movement or an inability to continue walking (Jankovic, 2008). This condition compromises gait stability and typically occurs during gait transitions, turning, navigating narrow spaces, or while performing dual tasks (Rahimpour et al., 2021). These various gait changes can also lead to asymmetric gait of both lower limbs, thereby affecting the balance of the body during walking (Rahimpour et al., 2021).

In PD AI gait recognition research, patients typically perform a series of gait tasks designed to assess the severity, progression, and mobility of the disease by analyzing the dynamic features of gait. The Regular Walking Task helps to capture the patient’s basic gait pattern, requiring the patient to walk at a normal pace on a flat surface (Ahmadi et al., 2021). This aims to assess the patient’s gait characteristics, such as step length, step frequency, stride length, swing, and support time, without any disturbances or obstacles. The gait acceleration or deceleration task requires patients to increase their speed to their maximum sustained speed or deliberately slow. These can be used to evaluate patients’ adjustment of gait when facing challenges, observing whether there is a phenomenon of shortened or unstable gait. In gait freezing tasks, patients are usually guided through certain situations, such as confined spaces (Almeida and Lebold, 2010), to observe whether the patient experiences freezing and to analyze the conditions and characteristics of its occurrence. Turning tasks typically require patients to walk along a particular curve or make turns in different directions within a defined area (King et al., 2022), while obstacle-crossing tasks require patients to cross an obstacle placed on the ground, such as a small step or an obstacle bar (Vitório et al., 2014). This task simulates scenarios that patients may encounter in their daily lives, estimating their gait stability and flexibility in dealing with obstacles. In dual-task-walking, patients need to perform other cognitive tasks (such as memorizing numbers, solving simple arithmetic problems, etc.) while walking (Kelly et al., 2012). Typically, patients show more pronounced gait abnormalities, particularly reduced stride length and unstable gait rhythm, when performing dual tasks (Salazar et al., 2017). These gait tasks play an important role in research into AI recognition of gait in PD. By collecting gait data from patients performing these tasks, researchers can use sensor technologies (e.g., inertial measurement units, accelerometers, gyroscopes, etc.) (Hubble et al., 2015) and AI algorithms to extract gait features, including spatiotemporal, kinematic, dynamic, electromyographic, sensor-based, and visual-based standing intervals, swing time, stride time, number of steps, gait velocity, stride length, walking speed, power spectral density, hip flexion, and plantar flexion offset (Sharma et al., 2022). These features provide the basis for building models to identify and predict gait abnormalities and assist clinicians in early diagnosis and monitoring of disease progression.

At present, there are datasets available for public use regarding the gait of PD. The “Gait in PD” (Hausdorff, 2008) database contains gait measurements, demographic information and disease severity from 93 patients and 73 healthy participants. The dataset includes recordings of the vertical ground reaction force of the participants, those who walked on a flat surface at their self-chosen pace for around 2 min. This information reflects the changes in pressure over time, as well as the activity time of each foot (such as stride time and swing time). The Gait in Neurodegenerative Disease Database (Hausdorff, 2000) records stride interval, swing interval, stance interval, double support interval information, and clinical information for each subject, including age, sex, height, weight, walking speed, and Hohn and Yahr scores for disease severity. Parkinson’s Freezing of Gait Prediction (The Michael J Fox Foundation, 2023) consists of three sub-datasets, each containing sensor and video recordings of patients’ frozen gait before and after medication, as well as their daily gait.

Machine learning and DL techniques have demonstrated substantial promise in leveraging gait analysis for the diagnosis and early detection of PD. These computational methods can detect subtle motor impairments by analyzing spatiotemporal gait parameters, often imperceptible to clinicians during routine examinations. The synergy of wearable sensor data, advanced signal processing, and AI models enables robust classification and staging of PD, including early-stage detection and symptom monitoring. For instance, the application of the support vector machine with linear decision boundaries has led to an overall classification accuracy of 95% (Alkhatib et al., 2020). Moreover, the reinforcement fine-tuning method not only facilitates binary classification for PD diagnosis but also attains an average accuracy of 96.4% ± 2.3% when assessing the severity of the disease (Varrecchia et al., 2021). In addition, there are specific tests for gait freezing in patients that play a role in helping to diagnose PD and can also predict gait freezing in time to prevent falls. Wearable inertial measurement units placed on limbs or embedded in smart insoles have been effectively used to collect gait data for input into AI models, achieving high sensitivity in identifying FoG episodes (Cluzel et al., 2025). The accuracy, sensitivity, and specificity of FOG prediction were all around 85% in patients receiving dopaminergic therapy (1 h after levodopa ingestion) and in those not receiving dopaminergic therapy (at least 12 h after levodopa discontinuation) (Borzì et al., 2021). Beyond detection, DL models also support disease monitoring and risk prediction. Multidomain analysis including gait, voice, and tapping through smartphones offers a scalable, non-invasive tool for PD screening, particularly in remote or resource-limited settings (Lim et al., 2025). Gait abnormalities, such as asymmetry, reduced stride length, and variability in cadence, have been effectively quantified using AI to track disease progression or response to medication or Deep Brain Stimulation (Priya et al., 2025). Furthermore, gait analysis has been proposed not just for diagnosis but also as a digital biomarker for fall prediction, severity scoring, and even to differentiate PD from atypical Parkinsonian syndromes (Terry and Alvarez-Vazquez, 2025). Based on postural instability and gait analysis, the application of an enhanced weighted voting ensemble method demonstrates high sensitivity but relatively low specificity when differentiating atypical parkinsonism from PD (Song et al., 2022). These advancements illustrate a shift toward personalized and continuous neurology, where machine intelligence enhances clinical decision-making. In summary, the integration of gait analysis with AI offers a promising frontier for improving the accuracy, objectivity, and timeliness of PD diagnosis. However, the biggest problem with gait analysis is that a complete standard collection procedure has not yet been established. Since most of the technicians are scientific researchers rather than experts with clinical experience, the methods of collecting patient gait vary from study to study. Moreover, the lack of data volume in the dataset is also an important aspect that affects the classification decision model (Sharma et al., 2022).

## 9 Discussion

This review primarily aims to explore the roles of motor symptoms and their corresponding characteristic manifestations of PD in AI-assisted diagnosis. We summarized the specific manifestations of facial expressions, eye movements, speech, handwriting, finger - tapping, and gait in PD, their applications in AI-assisted diagnosis, publicly accessible datasets, current limitations, and future development directions.

Generally speaking, research on PD recognition based on speech, handwriting, finger-tapping, and gait is relatively comprehensive, and the model recognition accuracy is quite high. In the realm of speech analysis, for instance, several studies delved into the unique pitch, rhythm, and articulation patterns associated with Parkinson’s patients (Faragó et al., 2023; Favaro et al., 2023b; Ma et al., 2021). Various publicly available datasets greatly facilitated the development of AI-based diagnostic models. Similarly, in handwriting, tremors, micrographia (abnormally small handwriting), and irregularities in stroke formation have been well-documented. These datasets have enabled the training of models to accurately identify signs of the disease from handwritten samples. In finger-tapping and gait analysis, a multi-class assessment of the disease progression degree can be accomplished, its speed, rhythm, and force exerted during the tapping motion can provide valuable insights into the progression of the disease (Kim et al., 2011; Rose et al., 2020). Gait analysis, on the other hand, focuses on parameters such as stride length, walking speed, and balance. However, despite these achievements, the accuracy of these assessment methods still requires improvement. There are still challenges in accounting for individual variations among patients, as well as the influence of external factors such as fatigue and environment. Regarding facial expression and eye movement recognition, perhaps due to the dynamic visual changes involved, the overall recognition accuracy still needs to be enhanced through further technological advancements when compared with other motor symptoms. Detecting these subtle changes in real time is a complex task, as facial expressions can be affected by emotional states and social context. Eye movement analysis, which includes saccades (rapid eye movements) and smooth pursuit, also presents challenges due to the high-speed and fine-grained nature of these movements. In the future, more in-depth studies are required to develop more accurate and robust AI-assisted diagnostic models. Additionally, the research of more advanced algorithms that can better handle the dynamic and complex nature of facial expressions and eye movements will be crucial for enhancing the recognition accuracy in these areas.

There remain numerous common issues that demand further attention. When it comes to raw data, a significant portion of studies is concentrated within a limited number of datasets, and the evaluation of the included patients and the recording of related information are incomplete. The quantity, quality, type, and representativeness of data play essential parts in model construction. For different types of motor symptoms, collecting raw data requires establishing a comprehensive standardized process to improve data quality and representativeness of typical features. When recruiting patients, a comprehensive evaluation should be carried out under the guidance of specialized doctors. It is necessary to make action tasks that can trigger specific manifestations and form expert consensus. Specific details such as the patient’s race, disease duration, and severity should be annotated to establish a large-scale, high-quality unified dataset, ensuring that the models are trained on a diverse and representative set of data, thus improving their generalization ability. In the aspect of visualizing dynamic information like eye movements and facial expression changes, technological advancements are still required to support the high-quality extraction and utilization of the necessary features. With the complexity of PD manifestations and the diversity of data sources, it is difficult to accurately extract valuable information from the raw data. Traditional data analysis methods may not be sufficient to handle the large-scale and high-dimensional data generated in modern research. Therefore, more advanced ML and AI algorithms should be introduced to dig out hidden patterns and relationships in the data. Moreover, there are certain challenges for AI to differentiating PD from atypical Parkinson-syndromes based solely on motor disorders. In such scenarios, AI may play a supportive role for doctors in detecting variations in the movement states (closed/open) of the same patient (Vasquez-Correa et al., 2019).

Currently, research on multimodal PD diagnosis based on large-scale and high-quality datasets and the development of wearable devices integrating multiple features are in high demand and represent a major trend in future research. Close cooperation between researchers, clinicians, and industry partners is essential. Researchers should work closely with clinicians to understand the actual needs in the clinical setting and develop practical solutions. Identification models should adapt to different application scenarios, such as community screening, home monitoring, and hospital-assisted diagnosis. Improving the real-time performance and usability of model recognition facilitates the early detection and diagnosis of the disease, enabling early intervention to delay disease progression.

## 10 Conclusion

This article reviews the role of the motor symptoms and corresponding characteristic manifestations of PD in AI-assisted diagnosis. At present, PD recognition technology based on speech, handwriting, Tap, and gait is relatively mature, but the extraction of dynamic features such as facial expressions and eye movements still needs to be strengthened. Establishing a standardized data collection process and a large-scale dataset containing multiple information labels is currently an urgent problem that needs to be solved. In the future, research on multimodal recognition models and wearable devices is a promising direction. These suggestions may provide better services for both patients and doctors and contribute to social healthcare.
